# Receptor Interacting Protein-2 Plays a Critical Role in Human Lung Epithelial Cells Survival in Response to Fas-Induced Cell-Death

**DOI:** 10.1371/journal.pone.0092731

**Published:** 2014-03-21

**Authors:** Mohd. Akhlakur Rahman, Kruthika Sundaram, Srabani Mitra, Mikhail A. Gavrilin, Mark D. Wewers

**Affiliations:** Dorothy M. Davis Heart and Lung Research Institute, Department of Internal Medicine, Division of Pulmonary, Allergy, Critical Care and Sleep Medicine, Wexner Medical Center, The Ohio State University, Columbus, Ohio, United States of America; Louisiana State University, United States of America

## Abstract

Lung epithelial cell death is critical to the lung injury that occurs in the acute respiratory distress syndrome. It is known that FasL plays a prominent role in this lung cell death pathway and may work in part through activation of the receptor interacting protein-2 (RIP2). RIP2 is serine/threonine kinase with a C-terminal caspase activation and recruitment domain (CARD). This CARD contains a highly conserved, predicted tyrosine phosphorylation site. Thus, involvement of tyrosine phosphorylation in the CARD domain of RIP2 may play a critical role in Fas-mediated apoptosis in the human lung immune system. To test this hypothesis, human lung epithelial cells (BEAS-2B) were induced to undergo cell death in response to the Fas agonist antibody CH11 with and without manipulation of endogenous RIP2 concentrations. We show that CH11 increases lung epithelial cell death in a dose-dependent manner as determined by LDH release and nuclear condensation. Fas-induced LDH release was inhibited by RIP2 knock-down. Reduced levels of RIP2 in BEAS-2B cells after treatment with RIP2 siRNA were confirmed by immunoblot. Overexpression of RIP2 in BEAS-2B cells synergized with Fas ligand-induced LDH release in a dose-dependent manner. Finally, mutation of the tyrosine phosphorylation site in CARD of RIP2 protected BEAS-2B cells from Fas ligand induced cell death. Thus RIP2's CARD tyrosine phosphorylation may represent a new therapeutic target to promote the survival of human lung epithelial cells in disorders that lead to acute lung injury and ARDS.

## Introduction

The Fas-Fas ligand (FasL) pathway has been demonstrated to contribute to severe epithelial damage that occurs in the acute respiratory distress syndrome (ARDS), a disease characterized by the death of lung epithelial cells with resultant loss of lung barrier function. Soluble FasL can be released as a biologically active, death-inducing mediator capable of inducing apoptosis of epithelial cells during acute lung injury [1]. This concept is supported by the finding that bronchoalveolar lavage fluid (BALF) from patients with ARDS can induce apoptosis of small airway epithelial cells, which are dependent on the Fas-FasL pathway [2]. Therefore, inhibiting this pathway may provide novel treatment strategies to ameliorate acute lung injury.

In this context, receptor interacting protein-2 (RIP2), a 61-kDa adaptor kinase, may play an important role in the host defense at barrier sites such as the lung and the gut. RIP2 also called RIP-like-interacting CLARP kinase (RICK) and caspase-recruitment domain (CARD)-containing IL-1β converting enzyme (ICE)-associated kinase (CARDIAK), is capable of inducing both NF-kB activation and cell death [3]–[7]. Disease associated polymorphisms in RIP2's upstream signaling partner, NOD2, have been described for early onset sarcoidosis [8], [9] and Crohn's disease [10]–[13]. Since RIP2 function may modulate the inflammatory and apoptotic function of epithelial cells we decide to investigate the role that RIP2 may play in modulating lung epithelial cells responses to FasL.

The surface Fas receptor (also known as CD95), a member of the tumor necrosis factor superfamily, is widely expressed and plays a critical role in the regulation and homeostasis of the immune system [14]. Activation of CD95 by FasL, a trimeric cell surface protein, leads to rapid induction of apoptosis [14]. The intracellular domain of CD95 and related death receptors contains a death domain that was originally described in the tumor necrosis factor receptor-1 [14]. The death domain of CD95 and tumor necrosis factor receptor-1 are responsible for signaling cell death [14].

It has been shown recently that RIP2 undergoes autophosphorylation on Tyr 474 (Y474) in its caspase recruitment domain (CARD) which is critical in its interaction with NOD2. This phosphorylation event is necessary for effective NOD2 signaling and is blocked in the presence of the most common Crohn's disease-associated NOD2 allele [15]. Of note, this RIP2 tyrosine site is conserved across vertebrate species [15].

Although RIP2 is best known as an upstream signaling kinase that is important for NFκB activation [3], [4], RIP2 has also been shown to be able to induce cell death in some settings. For example, overexpression of RIP2 induces apoptosis in cell lines such as human embryonic kidney cells and MCF7 breast cancer cells [4]. Several *in vitro* studies have shown that RIP2 can associate with a variety of other CARD-containing molecules through CARD–CARD interactions [3]–[5]. Interaction with the CARD containing cIAP-1 could also implicate RIP2 in modulation of apoptosis [4], [5]. cIAP-1 binds to and potently inhibits caspase activity. One mechanism suggested that RIP2 physically interacts with CLARP, a caspase-like molecule, a protein containing two death effector domains (DED) capable of binding to Fas-associated death domain (FADD) and caspase-8, mediated Fas-induced apoptosis [3].

However, how RIP2 plays a role in cells death is still largely unclear. In this study, we show that knock-down of RIP2 in human lung epithelial cells protects and overexpression of RIP2 synergizes with Fas-induced cell death in a dose-dependent manner. We also find that, upon Fas activation, RIP2 is inducibly serine/threonine and tyrosine phosphorylated. The phosphorylation to Tyr 474 (Y474) is required for maximal RIP2-induced cytotoxicity and maximal signaling synergy with Fas ligand. RIP2's CARD domain tyrosine phosphorylation site presents a new therapeutic target to promote the survival of human lung epithelial cells in disorders that lead to acute lung injury and ARDS.

## Materials and Methods

### Cells and reagents

Human lung epithelial cells (BEAS-2B) were purchased from ATCC (ATCC CRL9609). BEAS-2B cells were cultured in DMEM (MediaTech, Inc) supplemented with 10% heat-inactivated FBS (Atlanta Biologicals) and 1% penicillin-streptomycin (Invitrogen Life Technologies). Anti-Fas (human, activating), clone CH11 was purchased from Upstate Cell Signaling Solution, USA. Cells were regularly checked to confirm the absence of *Mycoplasma* contamination [16]. Cell culture medium was used for detection of LDH release as a cell death signature. Cell lysates were analyzed for proteins by immunoblots.

### Immunoprecipitation and immunoblot assay

Cells were lysed in RIPA buffer (50 mM Tris–HCl (pH7.5), 150 mM NaCl, 1 mM EDTA, 1 mM NaF, 1% NP-40 and 0.25% Na-deoxycholate) supplemented with complete protease inhibitor cocktail (Sigma), fresh 1 mM PMSF, 100 μM N-(methoxysuccinyl)-Ala-Ala-Pro-Val chloromethylketone) and 1 μM Na3VO4. Cell lysates were clarified by centrifugation at 16,000×*g* for 10 minute. Protein concentrations were determined using Bio-Rad Dc protein Lowry assay (Bio-Rad). For immunoprecipitation, mouse monoclonal anti-phosphotyrosine antibody; clone 4G10 from Millipore was used to pull-down total tyrosine phosphorylated protein in combination with protein G-agarose (Invitrogen). After SDS-PAGE gel separation, samples were transferred to a PVDF membrane, probed with the antibody of interest and developed by ECL (Amersham Biosciences). Rabbit polyclonal antibody against RIP2 was purchased from Cayman, and Phospho-RIP2 (Ser176) antibody from Cell Signaling.

### Cell death detection by quantification of lactate dehydrogenase (LDH) release in cell culture medium

Lactate dehydrogenase (LDH) release into cell culture medium was used as an indicator of cell death using NAD+ reduction assay (Roche Applied Science). Cells were plated in 12-well plate at the density 0.15×10^6^/ml and stimulated with CH11. Cell culture medium was collected, clarified by centrifugation, and used for LDH assay. Total LDH content in cells (positive control) was measured in cells lysed with Triton X-100 (1% final concentration). Cell culture medium alone was used as a blank and OD values were subtracted from readings of samples and positive control. LDH concentration in the medium was detected at wavelength 490 nm. Cell death was calculated by the formula: (cytotoxicity (%)  =  (sample/positive control) ×100), as described earlier [17].

### Construction of Expression Plasmids

RIP2 plasmid was a gift of J. Tschopp (University of Lausanne, Lausanne, Switzerland). Wild type human RIP2 was re-cloned in eukaryotic expression vector pCDNA3.1(+) and sequence verified. RIP2 site specific mutants (Y474A, Y474F and Y474D) were created by using standard site-directed mutagenesis protocol, where RIP2 plasmid was used as a template. Briefly, primers pairs (gatcatgaaagaggac**gc**tgaacttgttagtac and gtactaacaagttca**gc**gtcctctttcatgatc; catgaaagaggact**t**tgaacttgttagtac and gtactaacaagttca**a**agtcctctttcatg; gatcatgaaagaggac**g**atgaacttgttagtacc and ggtactaacaagttcat**c**gtcctctttcatgatc) were used in PCR reaction with Turbo-Pfu polymerase (Invitrogen) to substitute tyrosine 474 by alanine, phenylalanine or aspartic acid, respectively. PCR conditions were: 95°C for 1 min, then 25 cycles 95°C for 30 s and 68°C for 5 min with temperature decreasing rate at 1°C/s and increasing rate at 0.5°C/s, followed by the final amplification step at 68°C for 15 min. After PCR reaction was complete, the reaction mix was treated with DpnI restriction enzyme (NEB) for 1 h at 37°C to destroy methylated plasmid template and used to transform competent bacteria. Next morning bacterial colonies were inoculated into LB media and later plasmids were isolated by mini kit from Promega. RIP2 mutated constructs were confirmed by DNA sequencing.

### Real-time PCR

Cells were lysed in TRIzol (Invitrogen Life Technologies), and total RNA was converted into cDNA by ThermoScript RT system (Invitrogen Life Technologies). Quantitative PCR was done in the StepOne Plus machine using SYBR Green PCR mix (Applied Biosystems). The values of target genes were normalized to the values of two housekeeping genes, GAPDH and CAP-1, and expressed as relative copy number, as described previously [18]–[20].

### *siRNA* transfections

The following sequence of siRNA targeting the 3′UTR or endogenous RIP2: AAGAAGAAAUGUGUUUCAUAA [15] was used to knock down RIP2. Design of siRNA was optimized by making duplexes of 21-nucleotide (nt) RNA with 2-nt 3′ overhangs, custom sequence purchased from Sigma. BEAS-2B cells (0.15×10^6^ cells) were reverse transfected with 50 pmol scrambled control or RIP2 small interfering RNA (siRIP2) using lipofectamine 2000. Next day, the medium was replaced and cytotoxicity induced by FasL (CH11, 1.0 μg/mL) for an additional 24 hours. Cell supernatants and lysates were harvested and used for immunoblotting, quantitative PCR, and LDH assay.

### Statistics

All experiments were performed a minimum of three independent times (unless specified) and expressed as mean values ± SD. Comparison of groups for statistical difference were done using two-tailed unpaired Student's t test or ANOVA with post hoc measures where indicated. *p* value at *p*≤0.05 was considered significant.

## Results

### FasL-induces LDH release and nuclear condensation in bronchial epithelial cells

**Figure 1:**
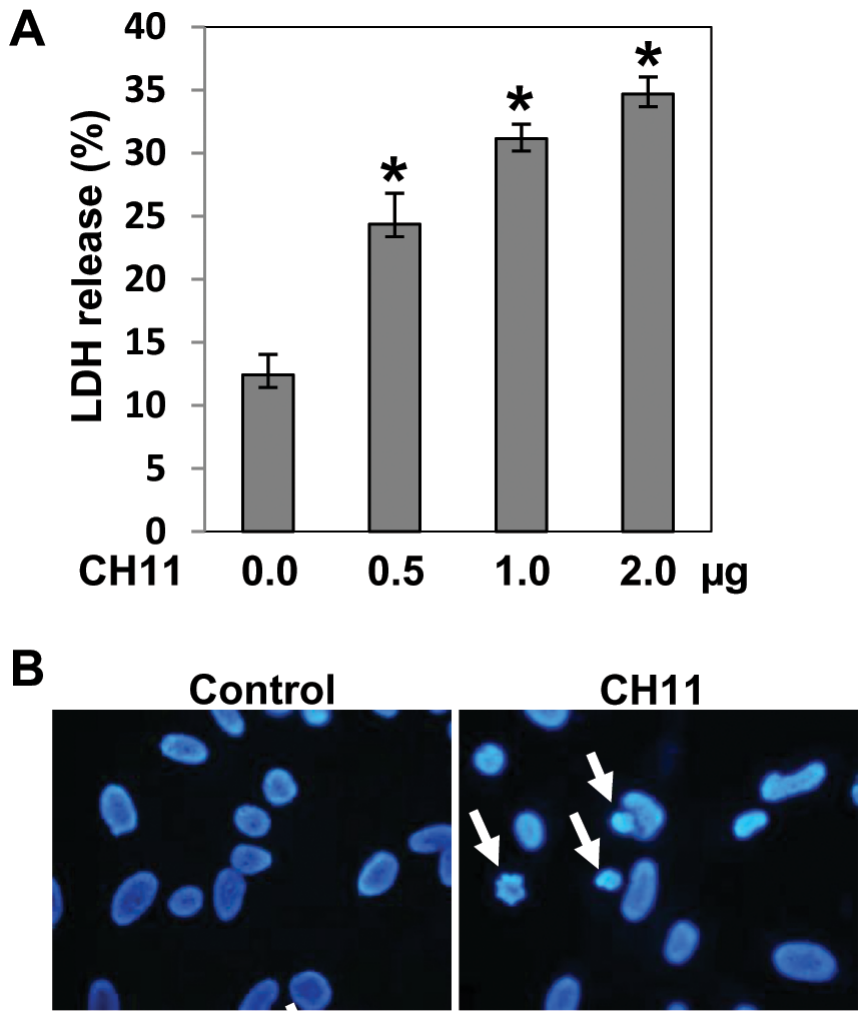
Fas ligand induced death of human lung bronchial epithelial cells. **A.** BEAS-2B cells were cultured 24 h in the presence of the FasL cross-linking antibody CH11 at the indicated concentrations. Cell death was detected by release of lactate dehydrogenase (LDH) in cell culture medium. Results are shown as mean ± SD of triplicate measurements. (* *p*<0.05 versus CH11 0.0 μg/ml), **B.** BEAS-2B cell death was imaged by DAPI nuclear staining using fluorescence microscopy in control or CH11 (1 μg/ml) after 24 h culture. The figure shows representative photomicrographs from two experiments with similar results.

The effect of Fas ligand (CH11) on BEAS-2B bronchial epithelial cell death was analyzed by two different methods, LDH release and DAPI staining for nuclear condensation. CH-11 significantly increased BEAS-2B cell death in a dose-dependent manner (**Fig 1A**). There was a significant increase in LDH release at doses of 1 μg/ml and higher, therefore, we used 1 μg/ml for the remainder of this study.

The LDH measure of cell death was confirmed by conventional DAPI staining which showed nuclear condensation characteristic of cell death (**Fig 1B**) and characteristic morphologic changes.

### RIP2 knock-down inhibits FasL-induced LDH release in bronchial epithelial cells

**Figure 2:**
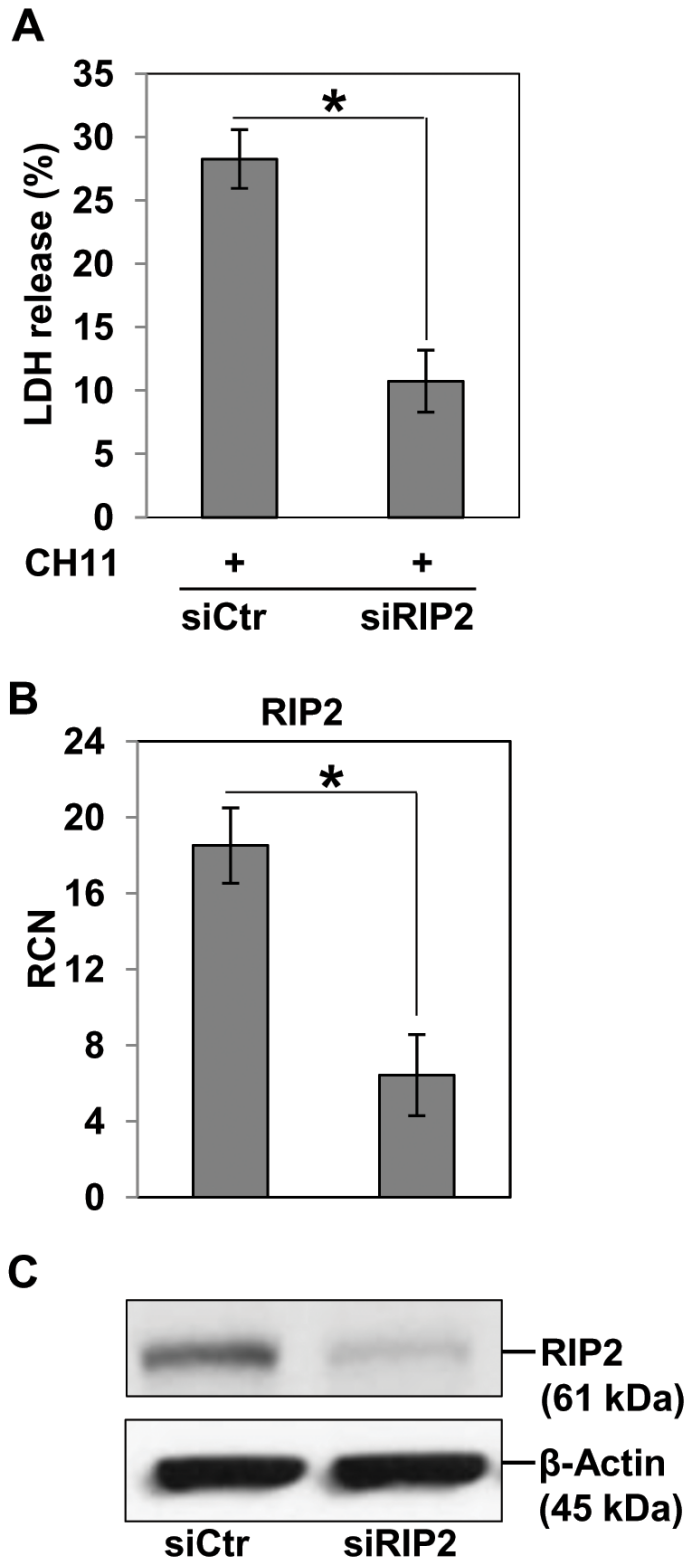
RIP2 knock-down ameliorate Fas ligand-mediated cytotoxicity in bronchial epithelial cells. **A.** BEAS-2B cells (1.5×10^5^ cells/well) in 12-wells plate were reverse transfected with 50 pmol scrambled control (siCtr) or RIP2 small interfering RNA (siRIP2) using lipofectamine 2000. On the following day the medium was replaced and cell treated with CH11, (1.0 μg/ml) for additional 24 h. Cell supernatants and lysates were harvested and used for immunoblotting, quantitative PCR, and LDH assay. **B.** BEAS-2B cells were harvested after 24 h of siRIP2, mRNA harvested, reverse transcribed to cDNA and quantified by RT-q (or quantitative) PCR for RIP2 expression and ***C,*** RIP2 protein expression differences between siCtr and siRIP2 BEAS-2B was determined by immunoblot with RIP2 specific antibody (at 1:200 dilution). β-actin was used as loading control. Results are shown as mean ± SD of triplicate measurements.The asterisk indicates a significant difference between siCtr and siRIP2-treated cells, as analyzed by Student's t-test (* *p*<0.05).

BEAS-2B cells were sensitive to apoptosis induced by Fas ligand, but were resistant to apoptosis after RIP2 knock-down. This finding suggested that RIP2 could promote apoptosis by interacting with signaling molecules of the CD95 death pathway. To determine if RIP2 expression is necessary for Fas-induced apoptosis, we investigated the effect of RIP2 on Fas-induced LDH release. **Fig 2A** shows that RIP2 knock-down but not control siRNA significantly inhibited Fas-induced LDH release. Reduced levels of RIP2 in BEAS-2B cells were confirmed by both qPCR (**Fig 2B**) and western blot (**Fig 2C**). These data indicate that the RIP2 is required for Fas-induced LDH release in lung epithelial cells.

### Overexpression of RIP2 synergizes with FasL-induced LDH release in bronchial epithelial cells

**Figure 3:**
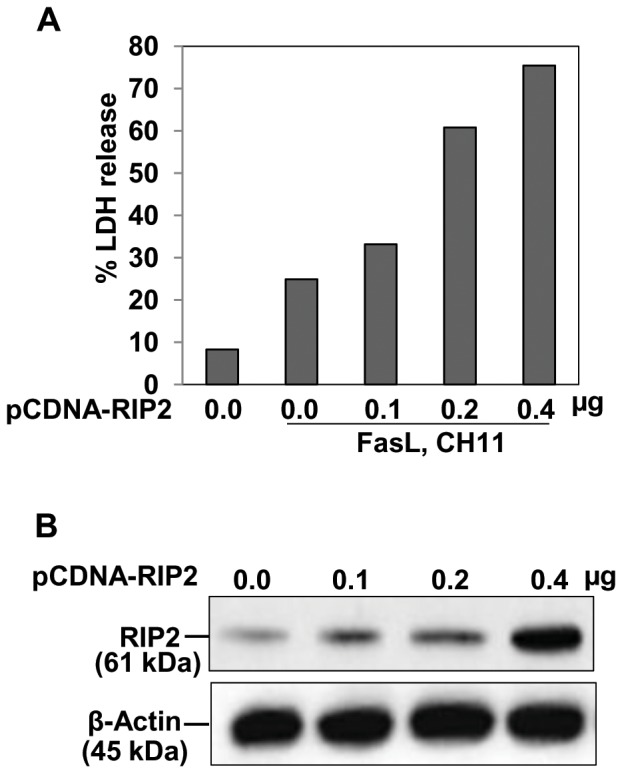
RIP2 overexpression augments on Fas ligand-induced cytotoxicity. BEAS-2B cells were transiently transfected with the indicated amounts of RIP2 expression vector. The total concentration of transfected DNA was kept constant by adding empty vector. After 24 h of transfection, medium was replaced and cells were treated with CH11 (1.0 μg/mL) for an additional 24 h. Cell supernatants and lysates were harvested and analyzed for **A.** LDH assay and **B.** immunoblotting of cell lysates for RIP2, respectively. The bars shown are expressed as means of duplicate independent experiments.

Because RIP2 knock-down inhibited Fas-induced LDH release, we also investigated the role of RIP2 in an over-expression system to confirm its role in modulating FasL-induced cell death. Human BEAS-2B cells were transfected with a eukaryotic expression vector encoding RIP2 and then treated with FasL. Overexpression of RIP2 synergized with FasL-induced LDH release in a dose-dependent manner (**Fig 3A**). Dose-dependent overexpression of RIP2 was confirmed by western blot with RIP2 specific antibody (**Fig 3B**).

### FasL phosphorylates RIP2 in bronchial epithelial cells

**Figure 4:**
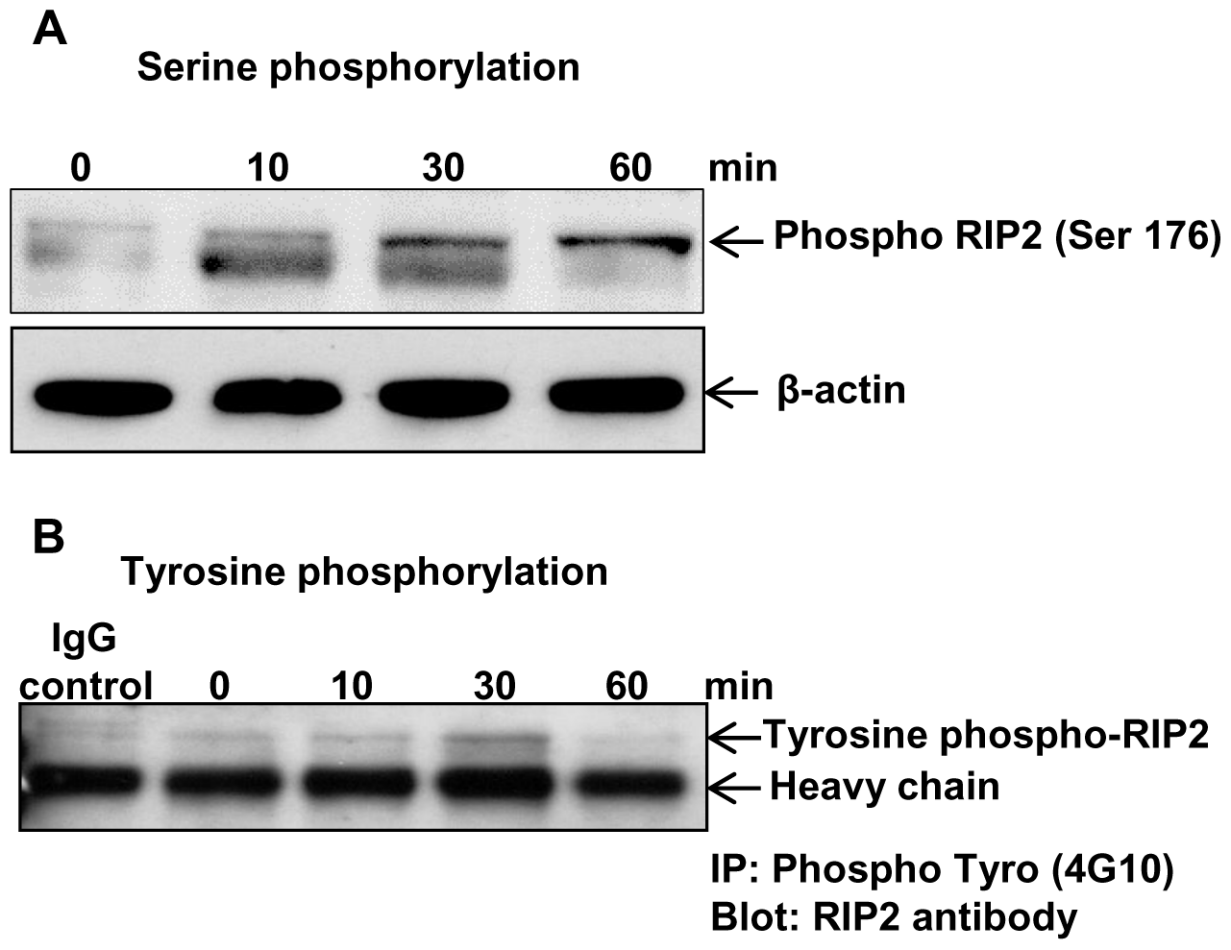
Fas ligand induces RIP2 phosphorylation. BEAS-2B cells were stimulated with CH11 (1.0 μg/mL), and lysates were harvested at the indicated time points. **A.** Expression of phospho-RIP2 (Ser176) in total cell lysates was analyzed by immunoblotting using phospho-RIP2 Ser176 specific antibody (at 1:500 dilution). The same samples were blotted with monoclonal antibodies against β-actin to confirm internal loading control. **B.** Tyrosine-phosphorylated RIP2 proteins were immunoprecipitated from the total cell lysates using phospho-tyrosine specific antibody 4G10 (at 1:500 dilution) and IgG control. Immunoblotting was performed with RIP2 specific antibody as indicated.

RIP2 is classically considered to be a serine kinase that itself undergoes significant post-translational modifications in response to activation of NOD2 [5], [15], [21]. Most of these changes involve serine or threonine phosphorylations, but it has recently been reported that RIP2 may also be tyrosine kinase that also undergoes tyrosine autophosphorylation [15]. Therefore, we attempted to determine whether RIP2 could be both serine or tyrosine-phosphorylated upon Fas activation. To determine this, BEAS-2B cells were first treated with the CH11 for different time intervals. We found that RIP2 could be both serine and tyrosine phosphorylated in response to Fas activation (**Fig 4**). Thus, in addition to the previous reports of RIP2 phosphorylation in response to NOD2 activation, the Fas-induced cell death signaling pathway also appears to induce RIP2 modifications.

### Mutation of Y474 causes a decrease in RIP2's ability to FasL-induced apoptosis

In an effort to understand the potential role of tyrosine phosphorylations in the regulation of RIP2's CARD/CARD interactions we searched for tyrosines predicted to undergo phosphorylation using NetPhos 2.0 predictions [22] in concert with BLAST analyses of vertebrate species conservation of these sites (ExPasy software) [23], [24]. One group of CARD containing proteins suggested that RIP2, NOD2 and ASC had predicted phosphotyrosine sites that were conserved across vertebrate species. The RIP2 tyrosine 474 corresponded with the recent documentation the NOD2 activation can induce phosphorylation at this site [15] (**Table 1**).

**Table 1:** Predicted tyrosine phosphorylations of CARD domains.*

Protein	CARD location	Tyrosine site	Prediction	Amino acid	Conserved
			(NetPhos 2.0)		
RIP2	carboxy	ED**Y**EL	0.97	474	yes
NOD2	amino	ED**Y**EG	0.96	71	yes
ASC	carboxy	EQ**Y**QA	0.91	146	yes

*Shown are tyrosine sites within the CARD of RIP2 **(r**eceptor **i**nteracting **p**rotein 2), NOD2 (**n**ucleotide-binding **o**ligomerization **d**omain-containing protein 2) and ASC (**a**poptosis-associated **s**peck-like protein containing a CARD) which are predicted by NetPhos 2.0 to be tyrosine phosphorylated and to have highly conserved sites across mammalian species.

**Figure 5:**
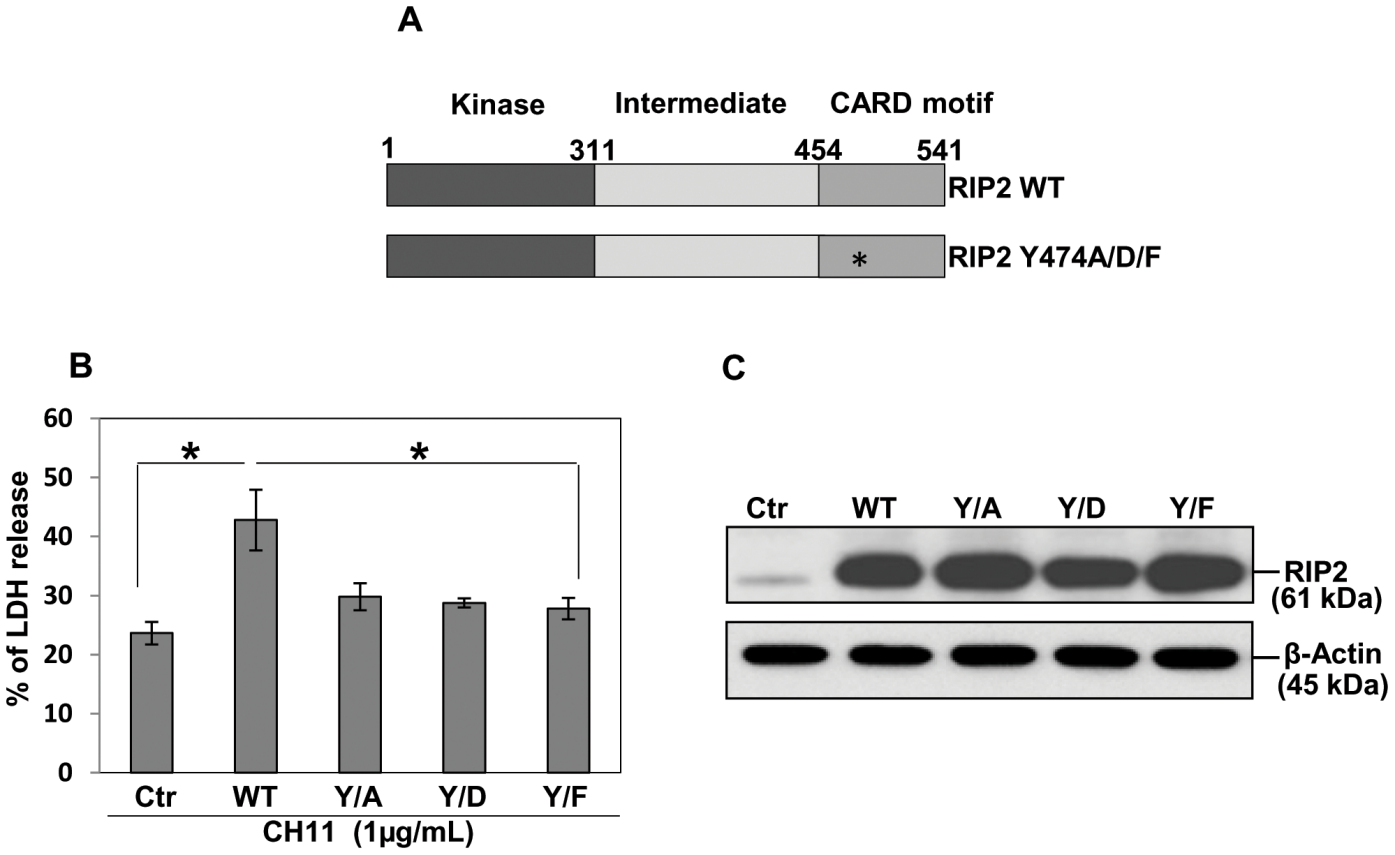
Mutating RIP2 tyrosine 474 decreases RIP2's ability to cell death induced by FasL. BEAS-2B cells were transiently transfected with wild type and CARD domain Y474 (Y/A, Y/D, or Y/F) mutants RIP2 expression vector. The total concentration of transfected DNA was kept constant by adding empty vector. After 24 h of transfection, medium was replaced and cells were treated with CH11(1.0 μg/mL) for an additional 24 h. **A.** Schematic representation of RIP2 and tyrosine mutation at 474 in CARD domain. Cell supernatants and lysates were harvested and analyzed for **B,** LDH assay and **C,** immunoblotting respectively. Asterisks indicate *p*<0.05 using one-way ANOVA with Tukey's post hoc comparison.

Since RIP2 contains a conserved tyrosine site in its CARD domain and we observed that RIP2 is tyrosine phosphorylated in response to Fas activation, we tested the hypothesis that mutation of the predicted tyrosine phosphorylation site in CARD domain of RIP2 might be capable of decreasing RIP2's role in FasL-induced apoptosis. To test this hypothesis, we generated site specific mutants of RIP2 (Y474) including Y474A, Y474D and Y474F (**Fig 5A**). Transient transfection assays showed that the Y474 mutation decreases RIP2's effect on cell death in response to Fas ligand (**Fig 5B**). Equal expression of transiently transfected RIP2 expression vectors (both wild type and mutant) were confirmed by immunoblots (**Fig 5C**). Collectively, these data support that it is the tyrosine phosphorylation site in the CARD domain of RIP2 that is responsible for Fas-induced cell death.

## Discussion

The main goal of this study was to determine whether the mechanism of bronchial epithelial cells death could be mediated by the involvement of RIP2 in response to FasL. Fas activation *in vivo* results in acute alveolar epithelial injury and lung inflammation, and may be important in the pathogenesis of acute lung injury [25]. Although the Fas/FasL pathway has been mainly implicated with respect to its death-inducing function, it also transduces signals through pathways that are still poorly defined.

In the current study, we have confirmed the involvement of RIP2 in Fas-mediated apoptosis, in addition to the well-known NOD2-RIP2 pathway. Presence of serine/threonine kinase and CARD domains of RIP2 suggested multifunctional roles of this adaptor cytosolic protein. In our experiments we have used immortalized normal human bronchial epithelial BEAS-2B cells as representative of human lung epithelium. Expression of Fas receptors in BEAS-2B cells has been reported, which support the involvement of Fas signaling pathway in the death processes of BEAS-2B cells [26]. Moreover, BEAS-2B cells have been shown to undergo apoptosis in response to FasL [27].

It has been reported that several factors contribute in Fas-signaling pathway. For example, IFN-γ enhances Fas-mediated apoptosis in A549 lung epithelial cells [28]. However, here we have shown that without addition of IFN-γ, treatment of BEAS-2B cells with only CH11 is dependent on RIP2. We have also analyzed the effect of IFN-γ in Fas-signaling pathway and observed that addition of IFN-γ followed by CH11 treatment in BEAS-2B cells enhances death through the up-regulation of Fas receptor expression (data not shown). But when we knocked-down RIP2 we did not see any effect of the loss of RIP2 in response to the addition of IFN-γ followed by FasL treatment, suggesting that IFN-γ utilizes a RIP2 independent pathway to induce death.

It has been revealed that RIP2 interacts with C-terminal domain of the CLARP [3], a caspase-related protein that interacts with FADD and caspase-8 and promote caspase-8 activity [29]–[34], and eventually to induce CD95 death pathway. Our data suggest that RIP2 protein synthesis in BEAS-2B cells is necessary for the Fas pathway, we show that loss of RIP2 protein in the cells reduces FasL-induced death (**Fig 2A**), indicating a specific role of RIP2 in Fas-mediated extrinsic pathway of apoptosis. In addition, RIP2 overexpression also enhances the overall apoptotic rate in response to FasL (**Fig 3A**). In other cell lines, such as the human embryonic kidney cells and MCF7 breast cancer cells, RIP2 overexpression also results in enhanced apoptosis [4]. These reports demonstrate that RIP2 overexpression acts as an inducer of caspase-8 activity. Using RT-qPCR and immunoblotting we validated the expression of RIP2 in knock-down and in overexpression experiments confirming that RIP2 is involved in Fas-mediated cell death (**Fig 2B, 2C** **,** **3B**).

Interestingly, caspase-1 does not appear to be involved in the cell death of BEAS-2B cells. A caspase-1 specific inhibitor did not block the RIP2 mediated apoptosis. This is noteworthy since it has been shown that CARD-CARD association between RIP2 and pro-caspase-1 may promote the processing of pro-caspase-1 into its active form [5]. However, later *in vivo* work has shown RIP2 to be dispensable for caspase-1 activation. [35].

Though RIP2 was originally classified as a serine/threonine kinase, in this study we focused on tyrosine phosphorylation in the CARD domain of RIP2 due to its highly conserved predicted phosphotyrosine site. Moreover, the limited abundance of tyrosine phosphorylation compared to phosphorylations on serine and threonine [36], [37], suggest that a tyrosine phosphorylation is likely to be biologically relevant. In this study, we show that RIP2 is both serine and tyrosine-phosphorylated after activation of the Fas pathway (**Figure 4**). These findings support that posttranslational modification of RIP2 in response to FasL is required to exert RIP2's critical role in lung epithelium death. Of note, AG126, a known phosphotyrosine kinase inhibitor, also inhibits CH11-induced BEAS-2B cell death (data not shown). These results support that the Fas receptor tyrosine phosphorylates of several intracellular proteins, involved in Fas receptor-induced cell death in both human and mouse [38].

Our results not only indicate that RIP2 overexpression enhances Fas-induced apoptosis but also show that mutating a conserved tyrosine site in the CARD domain of RIP2 inhibits apoptosis when overexpressed. Thus our finding invokes involvement of the CARD domain of RIP2 in apoptosis through the conserved tyrosine 474 site phosphorylation. Collectively, these results demonstrate that tyrosine phosphorylation in the RIP2 CARD domain is an important step in the generation of the Fas receptor-linked transmembrane death signal in lung epithelium. These finding also supports a previous report that RIP2 induced extensive cell death via its CARD motif [4].

Although the exact role of RIP2 in the activation of signaling molecules still remains to be elucidated, our results highlight the importance of maintaining adequate RIP2 protein levels to ensure the efficient transmission of Fas signals. We also analyzed signaling following Fas stimulation on RIP2 knock-down lung epithelial cells. BEAS-2B cells were stimulated with CH11 (1 μg/mL), and lysates were harvested at the indicated time points. Expressions of phospho-IκB-α and phospho-p38 were analyzed by immunoblotting. The results shown in Figure S1 indicate that under this condition, NF-κB and p38 activation did not take place completely in the RIP2 knocked-down cells as compared to siCtr cells. This data suggest that RIP2 is involved in Fas signaling at least in the NF-κB and p38 activation pathway.

Finally, it is important to comment upon some weaknesses of our approach. In order to be able to specifically modulate RIP2 expression levels we have focused exclusively on a human BEAS-2B cell model using the standard CH11 antibody mechanism of Fas activation. It is conceivable that human lung epithelial cells may behave differently *in vivo* when analyzed in the context of the complex lung milieu. However, despite these limitations we believe that our work supports a role for RIP2 in controlling lung epithelial cell responses to Fas pathways.

In summary, here we have shown that FasL induces lung epithelial cell death in a dose dependent manner. We have demonstrated that the loss of human RIP2 inhibits Fas-mediated lung epithelium cell death. We have also shown that RIP2 is serine and tyrosine phosphorylated in response to FasL, an event required for efficient Fas-mediated cell death. Moreover, we show RIP2 has been significantly involved in Fas-mediated cell death that is in part dependent upon 474 tyrosine phosphorylation at CARD domain. It is still not clear under which circumstances RIP2 tyrosine phosphorylation at 474 is necessary or facilitates Fas receptor mediated apoptosis. However, RIP2 tyrosine kinase activation seems to be required for the signaling induced by Fas, at least in some cellular systems such as human lung epithelium. Since FasL activation is known to play a role in lung injury, our findings suggest that RIP2's CARD tyrosine phosphorylation may be important in determining the fate of the lung epithelium in humans with lung injury.

## Supporting Information

Figure S1 RIP2 knock-down affects phosphorylation of effector signaling molecules in response to FasL. RIP2 knocked-down and control BEAS-2B cells were stimulated with CH11 (1 μg/mL), and lysates were harvested at the indicated time points. Expression of phospho-IκB-α and phospho-p38 were analyzed by immunoblotting using antibodies against the phosphorylated forms of IκB-α and p38. The same samples were blotted with monoclonal antibodies against β-actin. The proteins were detected by ECL.
(PDF)
