# Supplementary material for: Receptor Interacting Protein-2 Plays a Critical Role in Human Lung Epithelial Cells Survival in Response to Fas-Induced Cell-Death
Source: PLoS One. 2014 Mar 21;9(3):e92731. doi: 10.1371/journal.pone.0092731 (PMC3962444; doi:10.1371/journal.pone.0092731)
Supplement: Figure S1 — RIP2 knock-down affects phosphorylation of effector signaling molecules in response to FasL. RIP2 knocked-down and control BEAS-2B cells were stimulated with CH11 (1 μg/mL), and lysates were harvested at the indicated time points. Expression of phospho-IκB-α and phospho-p38 were analyzed by immunoblotting using antibodies against the phosphorylated forms of IκB-α and p38. The same samples were blotted with monoclonal antibodies against β-actin. The proteins were detected by ECL. (PDF) [file pone.0092731.s001.pdf]

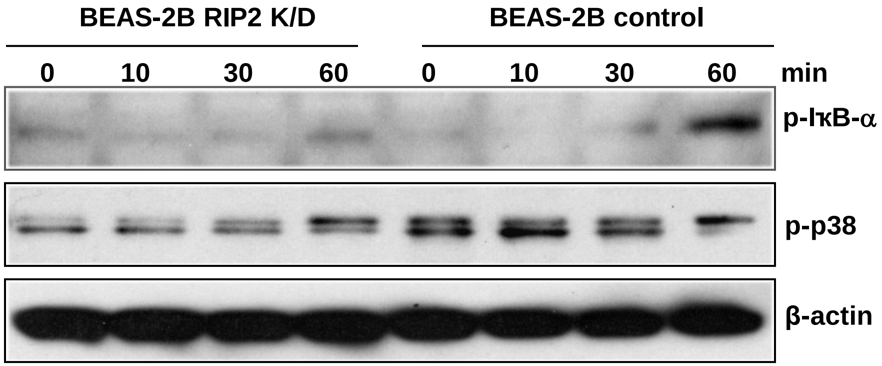

Figure S1. RIP2 knock-down affects phosphorylation of effector signaling molecules in response to FasL. RIP2 knocked-down and control BEAS-2B cells were stimulated with CH11 (1 µg/mL), and lysates were harvested at the indicated time points. Expression of phospho-IκB-α and phospho-p38 were analyzed by immunoblotting using antibodies against the phosphorylated forms of IκB-α and p38. The same samles were blotted with monoclonal antibodies against β-actin. The proteins were detected by ECL.
